# Supplementary material for: Molecular malaria surveillance using a novel protocol for extraction and analysis of nucleic acids retained on used rapid diagnostic tests
Source: Sci Rep. 2020 Jul 23;10:12305. doi: 10.1038/s41598-020-69268-5 (PMC7378824; doi:10.1038/s41598-020-69268-5)
Supplement: Supplementary file 2 — Supplementary information 2 [file 41598_2020_69268_MOESM2_ESM.pdf]

Molecular malaria surveillance using a novel protocol for extraction and analysis of nucleic acids retained on used rapid diagnostic tests

Etienne A. Guirou [a,b,¶], Tobias Schindler [a,b,¶,*], Salome Hosch [a,b], Olivier Tresor Donfack [c], Charlene Aya Yoboue [a,b], Silvan Krähenbühl [a,b], Anna Deal [a,b], Glenda Cosi [a,b], Linda Gondwe [a,b,d], Grace Mwangoka [d], Heavenlight Masuki [e], Nahya Salim [e], Maxmillian Mpina [a,b,d], Jongo Said [d], Salim Abdulla [d], Stephen L. Hoffman [f], Bonifacio Manguire Nlavo [g], Carl Maas [g], Carlos Cortes Falla [c], Wonder P. Phiri [c], Guillermo A. Garcia [c], Marcel Tanner [a,b] and Claudia Daubenberger [a,b,*]

[a] Department of Medical Parasitology and Infection Biology, Swiss Tropical and Public Health Institute, Basel, Switzerland

[b] University of Basel, Basel, Switzerland

[c] Medical Care Development International, Malabo, Equatorial Guinea

[d] Ifakara Health Institute, Bagamoyo Branch, United Republic of Tanzania

[e] Department of Paediatrics and Child Health, Muhimbili University of Health and Allied Sciences, Dar Es Salaam, Tanzania

[f] Sanaria Inc., Rockville, Maryland, USA

[g] Marathon EG Production Ltd, Malabo, Equatorial Guinea

¶These authors contributed equally to this work.

* Corresponding authors: tobias.schindler@swisstph.ch, claudia.daubenberger@swisstph.ch

Supplementary Figure S2. Detection of the gametocyte-specific transcript PF3D7_0630000 in blood on RDTs after three weeks of storage at RT.

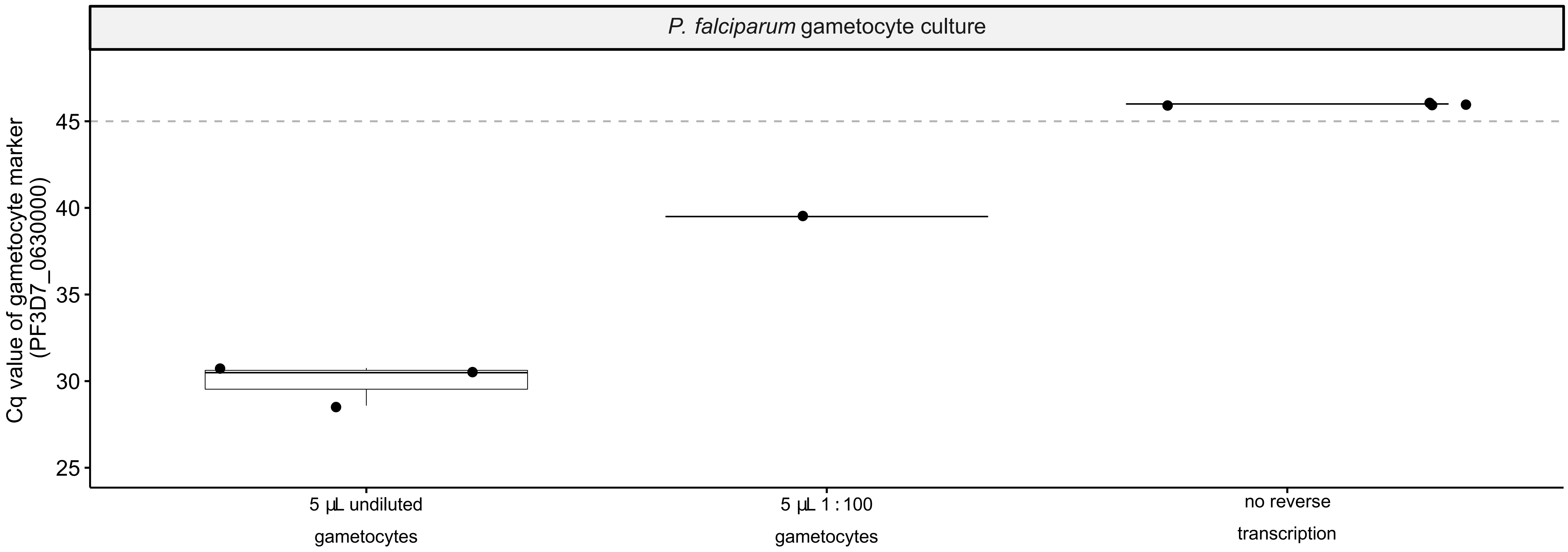

Extracted NA from 5 µL undiluted and 1:100 diluted culture amplified specifically the gametocyte marker, while the control without reverse transcription step did not result in amplification.
