# Supplementary material for: Molecular malaria surveillance using a novel protocol for extraction and analysis of nucleic acids retained on used rapid diagnostic tests
Source: Sci Rep. 2020 Jul 23;10:12305. doi: 10.1038/s41598-020-69268-5 (PMC7378824; doi:10.1038/s41598-020-69268-5)

Extraction of Nucleic Acids from RDTs (ENAR): step-by-step protocol

This is a detailed protocol describing the procedures for extraction of nucleic acids from malaria rapid diagnostic tests (RDTs) as evaluated in the publication:

Molecular malaria surveillance using a novel protocol for extraction and analysis of nucleic acids retained on used rapid diagnostic tests.

The extraction procedure was adapted from:
Zainabadi K et al. A novel method for extracting nucleic acids from dried blood spots for ultrasensitive detection of low-density Plasmodium falciparum and Plasmodium vivax infections. Malar J. 2017:16(1):377.

They provide an excellent and very detailed instruction on the preparation of the extraction buffer which should be followed.

IDENTIFICATION, SORTING AND TRACKING OF RDTs

The *RDTselect* app is a browser-based application which identifies barcode-labelled RDTs based on an input list containing all barcodes of a certain group. A single barcode can be scanned at a time with a mobile phone camera. The application identifies which group the sorted RDT belongs to.

The source code of the *RDTselect* app can be found at: *https://github.com/Sparclex/barcode-value-finder*

The *RDTallocator* app enables tracking of an individual RDT throughout the extraction process. The barcode is scanned with a mobile phone camera and the application allocates the associated RDT strip to the next available position in a 12-channel reservoir and subsequently 96-well plate.

The source code of the *RDTselect* app can be found at: *https://github.com/Sparclex/position-allocator*

REAGENTS AND MATERIALS

Table 1: Consumables

Article name	Supplier	Cat. number
12-channel multi-well reservoir	Axygen, Corning Inc, USA	RES-MW12-HP-SI
96-well collection plate	Omega bio-tec, Inc, USA	EZ9602-02
E-Z 96 DNA plate	Omega bio-tec, Inc, USA	BD96
DNA LoBind 96-well plate	Eppendorf AG, Germany	0030 603.303
Sterile, filtered pipette tips with volume range of 50-1200ul	various suppliers	

Table 2: Chemicals/Reagents

NB: if available, all chemicals and reagents should be designated as "Molecular Biology Reagents" and suitable for molecular biology applications.

Article name	Company	Cat number
DNA Elution Buffer	Zymo Research, USA	D3004-4-50
Ethanol	Sigma-Aldrich Chemie GmbH, Germany	51976-500ML-F
2-Propanol	Sigma-Aldrich Chemie GmbH, Germany	I9516-500ML
Molecular biology grade H_2O	Sigma-Aldrich Chemie GmbH, Germany	W4502-1L
Trizma hydrochloride buffer solution (pH 7.4)	Sigma-Aldrich Chemie GmbH, Germany	9313-1L
Guanine thiocyanate	Sigma-Aldrich Chemie GmbH, Germany	50981-500G
Triton X-100	Sigma-Aldrich Chemie GmbH, Germany	T8787-50ML
Sodium chloride	Sigma-Aldrich Chemie GmbH, Germany	S3014-500G
Hydrochloric acid, 6N	VWR Life Science, USA	E484-500ML
Ethylenediaminetetraacetic acid disodium salt solution (EDTA), 0.5 M	Sigma-Aldrich Chemie GmbH, Germany	E7889-100ML
2-Mercaptoethanol	Sigma-Aldrich Chemie GmbH, Germany	63689-100ML-F

Table 3: Equipment

Article name	Company	Reference number
Centrifuge for 96-well plates	-	
Multichannel pipets for volume range of 50-1200ul	Gilson/Eppendorf	PM14156/H41766H
Incubator (60 °C) with enough space for a rocking platform	-	
Rocking Platform/ lab shaker for 96-well plates (tolerating 60 °C).	-	

PREPARATION OF EXTRACTION BUFFERS

Lysis buffer / Wash 1 buffer

NB: Lysis buffer and Wash 1 are identical, with the exception that before use 0.5% 2-mercaptoethanol is added to the lysis buffer.

1. Add ~500 mL of water to a sterile 1 L bottle
2. Add all reagents as described in the table below:

**Concentrations used for lysis/wash 1 buffer**	**Amount for 1L of lysis/wash 1 buffer**
3M Guanidine thiocyanate	355 g Guanidine thiocyanate
16.7% 2-Propanol	167 mL 2-Propanol
2% Triton X-100	20 mL Triton X-100
10mM EDTA	20 mL 0.5 M EDTA solution
5mM Trizma hydrochloride buffer solution (pH 7.4)	5 mL Trizma hydrochloride buffer solution (pH 7.4)
0.1% Hydrochloric acid	1 mL 6M Hydrochloric acid

3. Shake until completely dissolved
4. Add up to 1000 mL with mol. biol. grade H_2O
5. Transfer 500 mL to a new bottle and label it with “Wash 1”.
6. Before use, 0.5 mL 2-mercaptoethanol is added to 100 mL lysis buffer.

Wash 2 buffer

1. Add all reagents to a sterile 1L bottle as described in the table below

**Concentrations used for wash 2 buffer**	**Amount for 1L of wash 2 buffer**
25% Ethanol	250 mL Ethanol
25% 2-Propanol	250 mL 2-Propanol
100mM Sodium chloride	20 mL 5M Sodium chloride solution
10mM Trizma HCl pH 7.4	10 mL Trizma hydrochloride buffer solution (pH 7.4)

2. Shake until completely dissolved
3. Add up to 1000 mL with mol. biol. grade H_2O

OPENING RDT CASSETTE AND REMOVING RDT STRIP

1. Open the RDT cassette, remove the RDT strip using sterile single-use forceps and transfer the strip into a 12-channel multi-well reservoir. Up to 96 RDT strips can be processed at the same time, using eight 12-channel reservoirs.
2. Add one positive control (RDT with known parasitemia, e.g. 5 µL blood with parasite density of 200 Pf/µL) and one negative control (lysis buffer only) to each extraction batch as controls.

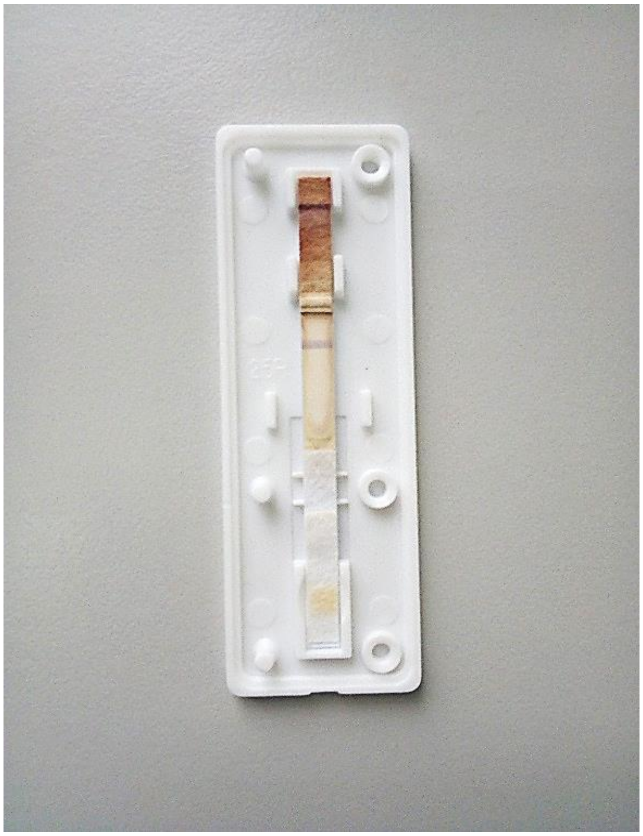

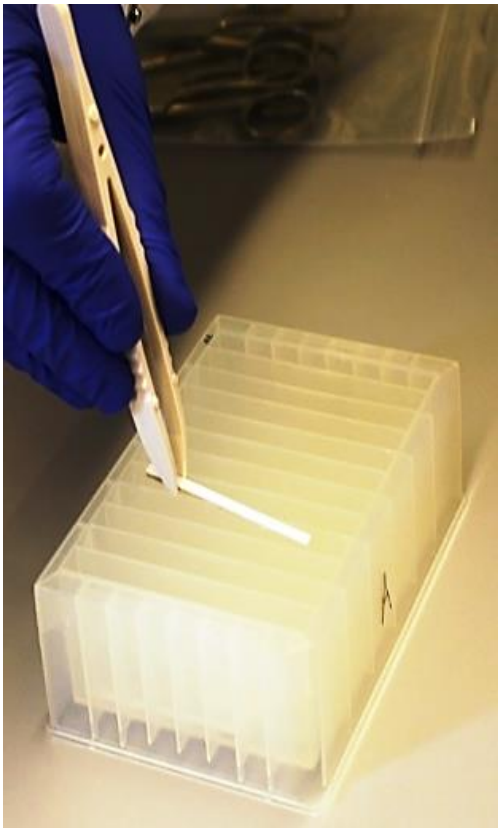

EXTRACTION OF NUCLEIC ACIDS FROM RDT STRIPS

1. Add **900 µL of Lysis Buffer** to each channel of the reservoir using a multichannel pipette. Make sure all RDT strips are completely submerged in the lysis buffer. Seal the reservoir with an adhesive aluminium foil.
2. Incubate the reservoirs **for 2 hours at 60 °C and shaking at 120 RPM.**
3. Secure an Omega E-Z 96 DNA plate (96 wells) to a 96-well collection plate with a tape.
4. Tilt the reservoir and transfer 900 µL of the supernatant (lysate) from the 12-channel reservoir to the E-Z 96 DNA Plate sitting on top of a 96-well collection plate.
5. Centrifuge the E-Z 96 DNA Plate (on top of the 96-well collection plate) at **3,500 RPM (2260 g) for 2 minutes.**
6. Discard the flow through and reuse the same 96-well collection plate.
7. Add **500µL of Wash 1** to the DNA plate and centrifuge at **3,500 RPM (2260 g) for 2 minutes**
8. Discard the flow through and reuse the same 96-well collection plate.
9. Add **500µL of Wash 2** to the DNA plate and centrifuge at **3,500 RPM (2260 g) for 3 minutes**
10. Discard the flow through and reuse the same 96-well collection plate.
11. Centrifuge the empty DNA plate at **3,500 RPM (2260 g) for 3 minutes** to completely dry the silica.
12. Place the E-Z DNA plate on top of a DNA LoBind 96-well plate for elution of nucleic acids.
13. Add **50µL** of pre-warmed **(60 °C)** Elution buffer to each well of the E-Z DNA plate to elute the nucleic acids.
14. Incubate the E-Z DNA plate at room temperature for **5 minutes.**
15. Centrifuge the E-Z DNA plate **3,500 RPM (2260 g)** for **3 minutes.**
16. Add an additional **25µL** of pre-warmed (60 °C) Elution buffer to each well of the E-Z DNA plate to elute the nucleic acids.

17. Incubate the E-Z DNA plate at room temperature for **5 minutes.**

18. Centrifuge the E-Z DNA plate **3,500 RPM (2260 g) for 3 minutes.**

19. Discard the E-Z DNA Plate and seal the DNA LoBind 96-well plate containing the eluted nucleic acids using aluminum foil.

20. Store the DNA LoBind 96-well plate containing the nucleic acids at **-20 °C or -80 °C**.

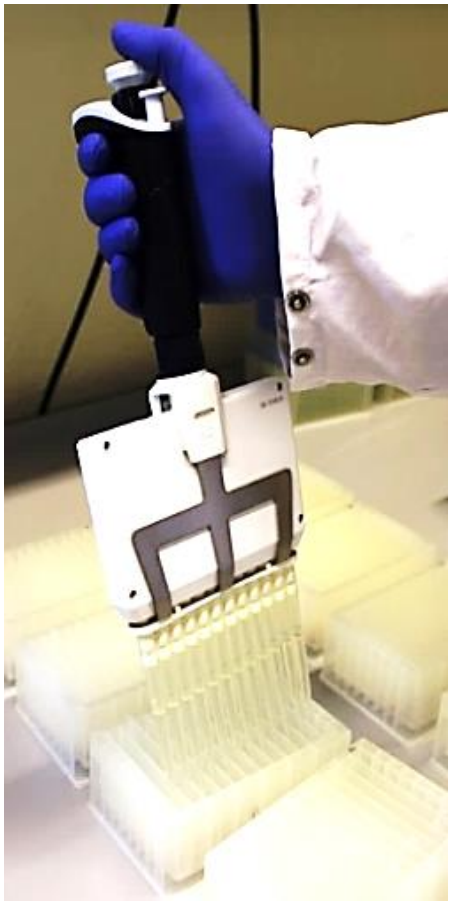

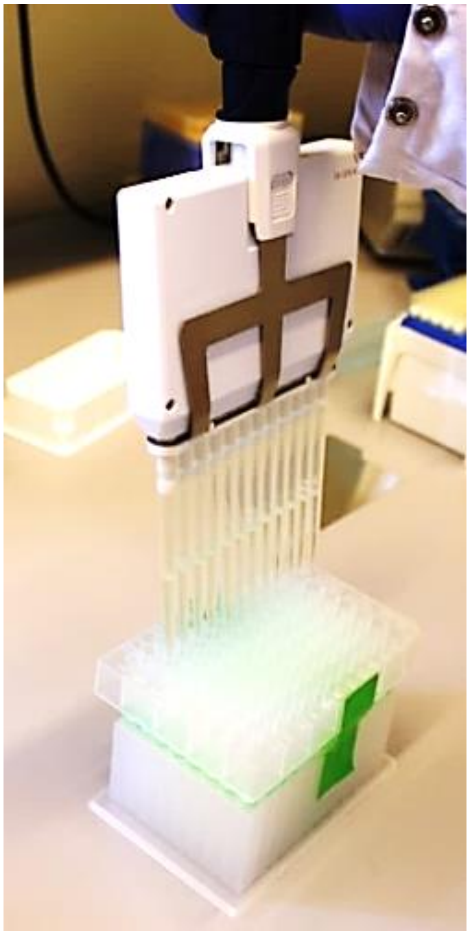

Supplement: Supplementary file 3 — Supplementary information 3 [file 41598_2020_69268_MOESM3_ESM.pdf]
