# Supplementary material for: Molecular malaria surveillance using a novel protocol for extraction and analysis of nucleic acids retained on used rapid diagnostic tests
Source: Sci Rep. 2020 Jul 23;10:12305. doi: 10.1038/s41598-020-69268-5 (PMC7378824; doi:10.1038/s41598-020-69268-5)
Supplement: Supplementary file 4 — Supplementary information 4 [file 41598_2020_69268_MOESM4_ESM.pdf]

# Molecular malaria surveillance using a novel protocol for extraction and analysis of nucleic acids retained on used rapid diagnostic tests

Etienne A. Guirou [a,b,¶], Tobias Schindler [a,b,¶,*], Salome Hosch [a,b], Olivier Tresor Donfack [c], Charlene Aya Yoboue [a,b], Silvan Krähenbühl [a,b], Anna Deal [a,b], Glenda Cosi [a,b], Linda Gondwe [a,b,d], Grace Mwangoka [d], Heavenlight Masuki [e], Nahya Salim [e], Maxmillian Mpina [a,b,d], Jongo Said [d], Salim Abdulla [d], Stephen L. Hoffman [f], Bonifacio Manguire Nlavo [g], Carl Maas [g], Carlos Cortes Falla [c], Wonder P. Phiri [c], Guillermo A. Garcia [c], Marcel Tanner [a,b] and Claudia Daubenberger [a,b,*]

[a] Department of Medical Parasitology and Infection Biology, Swiss Tropical and Public Health Institute, Basel, Switzerland

[b] University of Basel, Basel, Switzerland

[c] Medical Care Development International, Malabo, Equatorial Guinea

[d] Ifakara Health Institute, Bagamoyo Branch, United Republic of Tanzania

[e] Department of Paediatrics and Child Health, Muhimbili University of Health and Allied Sciences, Dar Es Salaam, Tanzania

[f] Sanaria Inc., Rockville, Maryland, USA

[g] Marathon EG Production Ltd, Malabo, Equatorial Guinea

¶These authors contributed equally to this work.

* Corresponding authors: tobias.schindler@swisstph.ch, claudia.daubenberger@swisstph.ch

Table S1. Summary of published studies using DNA extracted from RDTs for molecular analysis of malaria parasites.

| **Publication/Year** | **Veron** et al. **2006** | **Ishengoma** et al. **2011** | **Cnops** et al. **2011** | **Morris** et al. **2013** | **Papa-Mze** et al. **2015** | **Nabet** et al. **2016** | **Ndiaye** et al. **2017** | **Boyce** et al. **2018** | **Robinson** et al. **2019** | **Nag** et al. **2019** | **Nguyen** et al. **2019** |
|---|---|---|---|---|---|---|---|---|---|---|---|
| Origin of RDTs | French Guiana | Tanzania | Belgium | Tanzania | Senegal, Comoros Islands | Mali | Senegal | Uganda | Kenya | Guinea-Bissau | Gabon |
| Storage time and temperature | 6-36 months at room temperature | 28-65 days at room temperature | 1 week – 16 month at room temperature | 1-9 months at room temperature | not specified | 14 months at room temperature | <1 month at RT | 2 years at room temperature | 14 months at −20 °C | 3-18 months at room temperature | 0-6 months at room temperature |
| Number of samples for clinical evaluation | 40 | 165 | 121 | 855 | 204 | 134 | 572 | 299 | 141 | 2488 | 669 |
| Brand of RDT used for clinical evaluation | Optimal®, ICT Now® | ParaHIT®f, Paracheck Pf® | 12 different RDT brands were assessed, OptiMAL and SD BIOLINE Malaria Ag P.f® and P.f/Pan® were used to determine sensitivity | Paracheck-Pf, SD BIOLINE Malaria Ag P.f® and P.f/Pan® | SD BIOLINE Malaria Ag P.f® and P.f/Pan® | SD BIOLINE Malaria Ag P.f® and P.f/Pan® | SD BIOLINE Malaria Ag P.f® and P.f/Pan® | SD BIOLINE Malaria Ag P.f® and P.f/Pan® | SD BIOLINE Malaria Ag P.f® and P.f/Pan® | not specified | VIKIA® Malaria Ag Pf/Pan, Paracheck Pf, SD BIOLINE Malaria Ag P.f® and P.f/Pan® |
| Part of RDT used for clinical evaluation | proximal part | proximal part | proximal part | distal part | proximal part | entire strip | proximal | distal part | central part | not specified | proximal part |
| Extraction method | Phenol/chloroform extraction performed better than Chelex method or QIA amp DNA mini blood kit | Chelex method | Simple boil and spin method outperformed QIAamp DNA mini kit | Chelex method outperformed simple boil and spin method and ABI-based extraction | QIAamp DNA Mini kit | Automated extraction using NucliSENS EasyMAG instrument | Chelex method | Chelex method | Chelex method and robotic extractions using QIAsymphony gave comparable results | Chelex method | QIA amp DNA mini blood kit |
| Parasite detection | 18S rDNA nested PCR | 18S rDNA nested PCR | 18S rDNA qPCR | 18S rDNA nested PCR, cytochrome b nested PCR, and 18S rDNA qPCR | Nested PCR and HRM-qPCR for genotyping | 18S rDNA qPCR | pfcrt PCR-SSOP-ELISA | pfldh qPCR | pgmet-tRNA qPCR | 18S rDNA PCR | 18S rDNA qPCR |
| Quantification of parasites | no | no | no | no | no | yes | no | no | yes | no | yes |
| Analytical sensitivity | 50 Pf/µL with 15 µL diluted blood from malaria patient | 1 Pf/µL with 10 µL diluted blood from malaria patient | 0.02 Pf/µL with diluted blood from malaria patient | 2 Pf/µL with 5 µL diluted *P. falciparum* culture | not specified | not specified | not specified | not specified | 0.05 Pf/µL based on detection on a single RDT | not specified | 70 Pf/µL with 5 µL diluted blood from malaria patient |
| Clinical evaluation (detection rates) | 94.3% detection rate for samples with parasite densities ≥ 400 parasites/µL | 91.0% detection rate for samples positive by microscopy | 100% detection rate of clinical samples with parasite densities ≥ 1 parasites/µL | No difference in detection rates compared to DBS samples from febrile patients | 98.5% amplification rate for genotyping among positive RDTs | 79.8% detection rate among positive RDTs collected from symptomatic febrile patients | 94.7% detection rate among positive RDTs | 96.0% amplification rate among RDTs from patients with severe malaria | 81.25% agreement with DBS from children with positive RDT s | 74.0% detection rate among positive RDTs from suspected malaria cases | 96.6% detection rate during screening for a clinical trial |
| Non-*P. falciparum* species detected by NAT | P. vivax | no | P. vivax, P. ovale, P. malariae | no | no | no | no | no | no | no | no |
